# Genetic Resources and Vulnerabilities of Major Cucurbit Crops

**DOI:** 10.3390/genes12081222

**Published:** 2021-08-07

**Authors:** Rebecca Grumet, James D. McCreight, Cecilia McGregor, Yiqun Weng, Michael Mazourek, Kathleen Reitsma, Joanne Labate, Angela Davis, Zhangjun Fei

**Affiliations:** 1 Department of Horticulture, Michigan State University, East Lansing, MI 48824, USA; 2 USDA, ARS, Crop Improvement and Protection Research Unit, Salinas, CA 93905, USA; lettucemelon@gmail.com; 3 Department of Horticulture and Institute of Plant Breeding, Genetics & Genomics, University of Georgia, Athens, GA 30602, USA; cmcgre1@uga.edu; 4 USDA-ARS Vegetable Crops Research Unit, Madison, WI 53706, USA; yiqun.weng@wisc.edu; 5 School of Integrative Plant Science, Plant Breeding & Genetics Section, Cornell University, Ithaca, NY 14853, USA; mm284@cornell.edu; 6 North Central Regional Plant Introduction Station, Iowa State University, Ames, IA 50014, USA; kathleen.reitsma@usda.gov; 7 Plant Genetic Resources Unit, United States Department of Agriculture, Agricultural Research Service, Geneva, NY 14456, USA; joanne.labate@ars.usda.gov; 8 Sakata Seed America, Inc., Woodland, CA 95776, USA; angela.davis.phd1@gmail.com; 9 Boyce Thompson Institute, Cornell University, Ithaca, NY 14853, USA; zf25@cornell.edu

**Keywords:** Cucurbitaceae, *Cucumis*, *Citrullus*, *Cucurbita*, germplasm, ex situ conservation, genebanks

## Abstract

The Cucurbitaceae family provides numerous important crops including watermelons (*Citrullus lanatus*), melons (*Cucumis melo*), cucumbers (*Cucumis sativus*), and pumpkins and squashes (*Cucurbita* spp.). Centers of domestication in Africa, Asia, and the Americas were followed by distribution throughout the world and the evolution of secondary centers of diversity. Each of these crops is challenged by multiple fungal, oomycete, bacterial, and viral diseases and insects that vector disease and cause feeding damage. Cultivated varieties are constrained by market demands, the necessity for climatic adaptations, domestication bottlenecks, and in most cases, limited capacity for interspecific hybridization, creating narrow genetic bases for crop improvement. This analysis of crop vulnerabilities examines the four major cucurbit crops, their uses, challenges, and genetic resources. ex situ germplasm banks, the primary strategy to preserve genetic diversity, have been extensively utilized by cucurbit breeders, especially for resistances to biotic and abiotic stresses. Recent genomic efforts have documented genetic diversity, population structure, and genetic relationships among accessions within collections. Collection size and accessibility are impacted by historical collections, current ability to collect, and ability to store and maintain collections. The biology of cucurbits, with insect-pollinated, outcrossing plants, and large, spreading vines, pose additional challenges for regeneration and maintenance. Our ability to address ongoing and future cucurbit crop vulnerabilities will require a combination of investment, agricultural, and conservation policies, and technological advances to facilitate collection, preservation, and access to critical Cucurbitaceae diversity.

## 1. Introduction

**Figure 1:**
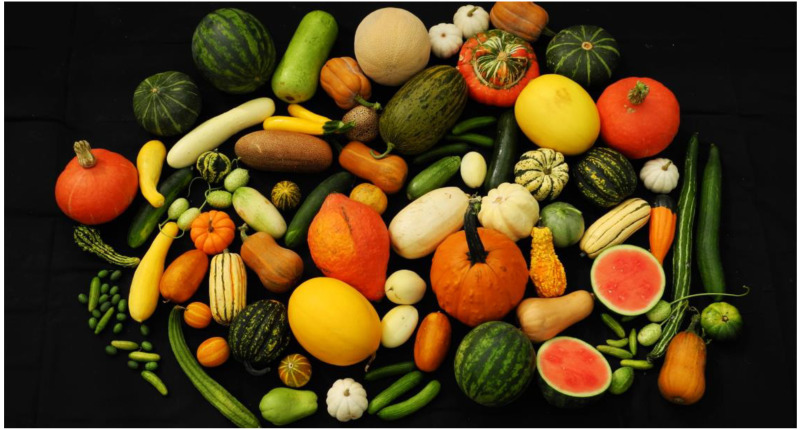
Cucurbit crops exhibit extensive diversity in fruit size, shape, and color.

A limited number of plant families supply the majority of crops that form the basis for human diets. Among these is the Cucurbitaceae family, providing an array of nutritional, flavorful, and colorful crops including watermelons (*Citrullus lanatus* (Thunb.) Matsum. and Nakai), melons (*Cucumis melo* L.), cucumbers (*Cucumis sativus* L.), and pumpkins and squashes (*Cucurbita* spp.). These crops, which are primarily consumed for fruits and seeds, are noted for remarkable diversity in fruit size, shape, and color, providing both culinary and aesthetic value (Figure 1). Other less widely cultivated cucurbits such as bitter gourd (*Momordica charantia* L.), bottle gourd (*Lagenaria siceraria* (Molina) Standley), wax gourd (*Benincasa hispida* (Tunb.)), snake gourd (*Trichosanthes* spp.), and sponge and ridge gourd (*Luffa* spp.), are also eaten as vegetables or seeds, used as sources of oils or medicines, or purposed as sponges or containers [1].

**Figure 2:**
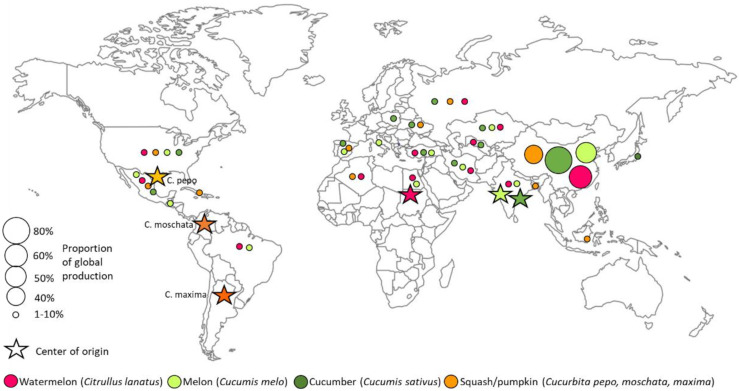
Regions of production and origins of domestication of major cucurbit crops: watermelon (*Citrullus lanatus*), melon (*Cucumis melo*), cucumber (*Cucumis sativus*), pumpkins/squashes (*Cucurbita pepo, moschata, maxima*). Circle size reflects relative proportion of worldwide production.

Production data for the predominant cucurbit crops are maintained by the Food and Agriculture Organization of the United Nations (FAO) (http://www.fao.org/faostat/en/#data/QC) (Accessed 10 March 2021) [2]. In 2019, worldwide cucurbit production included 100.4 Mt (megatonnes) for watermelon, 87.8 Mt for cucumber, 27.5 Mt for other melons, and 22.9 Mt for pumpkins and squashes (Table 1). While more than 100 countries (105–132) produce each of these crops in sufficient quantity to be recorded, in each case, the top 12 producing countries contribute greater than 70% of the global yield (ranging from 70.3% for pumpkins and squashes to 91.6% for cucumbers). China alone produces greater than one-third of worldwide production for each of these crops, contributing 36.7% of pumpkin and squash production; 49.1% of melons; 60.5% of watermelon; 80.1% of cucumber (Table 1, Figure 2). The other cucurbit crop recorded by the FAO is the melon seed (egusi), with a worldwide production of 1.0 Mt, of which 94% is produced in Africa, especially Nigeria with 60% of total production. Egusi seed can be produced from several different species, most commonly from *Citrullus mucosospermus* [3].

Human dependence on a limited number of crops is intensified by increasing population and demand for caloric requirements and nutritional quality. Advances in agricultural technologies (e.g., fertilizers, mechanization, breeding) over the past century have led to increased capacity for food production, especially for staple grain crops. While the increased productivity has greatly reduced the frequency and intensity of severe starvation events, widespread inadequacies remain for essential vitamins, minerals, and other nutritional components [4]. Horticultural crops, such as those in the Cucurbitaceae, have important roles to play in meeting these needs. Currently, our capacity to produce crops, including cucurbits, is subject to numerous vulnerabilities imposed by abiotic and biotic stresses and narrow genetic bases limiting adaptive potential. As we look to the future, there is reason to believe that the impacts of these stresses, both biotic and abiotic, will only magnify due to the compounding effects of increased demand combined with impacts of climate change, habitat destruction, and genetic erosion [5,6,7]. Our ability to meet these current and future challenges will require a concerted deployment of agricultural and conservation policies, technological advances, and preservation of, and access to, biological diversity resulting from adaptive evolution of crops and their progenitors in a wide range of environments.

Among the traits associated with domestication and crop improvement for cucurbits are reduced seed dormancy, increased fruit size and sweetness, reduced bitterness and acidity, and loss of daylength dependence for flowering habit [8]. As has been observed for many crops, genomic analyses of cucumber, melon, and watermelon show evidence of numerous selection sweeps. Reduced nucleotide diversity was observed in association with key domestication and breeding traits such as fruit size, nonbitter fruits, and flowering time in cucumber [9,10]. In melon, independent, essentially nonoverlapping sets of domestication sweeps were identified in ssp. *melo*, versus ssp. *agrestis* in association with fruit mass (weight, diameter, flesh thickness), loss of bitterness, and reduced acidity [11,12]. Similarly, in watermelon, allele fixation was observed for nonbitterness and carbohydrate metabolism and transport traits influencing flesh sweetness [13]. There is also evidence to suggest that genetic bottlenecks arising from domestication are particularly severe for cucurbits relative to values observed for grain crops [9]. It has been hypothesized that, in contrast with grain crops, the vining growth characteristics of cucurbits, which consume a large land area per plant and a large number of seeds per fruit, have reduced the diversity of seed sources maintained by human communities. 

Subsequent to domestication, cucurbits were transported and cultivated throughout the world. Production in tropical, subtropical, and temperate climates with a wide range of temperature, humidity, and rainfall conditions was accompanied by local adaptation and diversifying selection [8]. The resulting secondary sources of diversity include Africa for melon [14] and southern Africa for watermelon [15,16]. Cucumber germplasm diverged as it moved from India both eastward (to east Asia), and westward (to central/west Asia and beyond) [9,17,18,19,20]. In recent times, multiple factors, including the adoption of modern cultivars and the loss of habitat have led to the erosion of diversity for all crops. International consolidation of the seed industry, as has been observed over the past few decades, may accelerate this trend for modern cultivars. At the same time, in situ preservation of landraces is in danger from environmental challenges, including those that may result from climate change. For example, in Rajasthan, where melons are more or less naturalized, they are solely dependent upon Monsoon rains. In 1992, the Indo-US Cucumis expedition noted a complete loss of certain cucumber landraces in northern Rajasthan due to repeated drought [21]. 

One of the primary strategies to counteract loss and preserve genetic diversity has been the establishment of ex situ germplasm banks, typically housing seeds collected from around the world with an emphasis on centers of diversity. The importance of these collections as a critical resource for future world food security, along with the need to share genetic information and germplasm, has been formally recognized in the United Nations International Treaty on Plant Genetic Resources for Food and Agriculture (2001) [22]. A central clearinghouse for this information, GeneSys (https://www.genesys-pgr.org, accessed on 6 March 2021) [23], was established in 2011 as an outcome of this recognition. Among the sources of information included in the GeneSys database are the European Cooperative Programme for Plant Genetic Resources (EURISCO), Consultative Group on International Agricultural Research (CGIAR), and the United States Department of Agriculture National Plant Germplasm System (USDA NPGS). The primary cucurbit holdings documented in GeneSys are listed in Table 2. 

Other countries and germplasm repositories with significant cucurbit holdings not currently documented in GeneSys include those in China (China National Vegetable Germplasm Bank, Institute of Vegetables and Flowers at the Chinese Academy of Agricultural Sciences (IVF-CAAS)), Japan (National Agriculture and Food Research Organization (NARO); https://www.naro.affrc.go.jp/archive/nias/eng/genresources/index.html) (Accessed 20 April 2021), Russia (the Vavilov Institute of Plant Industry; http://www.vir.nw.ru) (Accessed 20 April 2021), India (Indian Council of Agricultural Research (ICAR)–National Bureau of Plant Genetic Resources (NBPGR) (http://www.nbpgr.ernet.in/) (Accessed 20 April 2021)), Korea (National Agrobiodiversity Center, (http://genebank.rda.go.kr/) (Accessed 15 June 2021)), Uzbekistan (the Uzbek Research Institute of Plant Industry, the Uzbek Research Institute of Vegetables, Melons, and Potato, the Karakalpak Research Institute of Agriculture, Tashkent, and the World Vegetable Center (https://avrdc.org, http://seed.worldveg.org/) (Accessed 15 June 2021)). Seeds of cucurbit species also have been deposited for long term preservation in the Svalbard Global Seed Vault: *Cucumis-* 3796 accessions of 22 species from 16 depositors, including 1098 *C. melo*, and 2515 *C. sativus*; *Citrullus*- 526 accessions of 7 species from 11 depositors, including 472 *C. lanatus*; *Cucurbita-* 1684 accessions of 16 species from 18 depositors, including 254 *C. maxima*, 579 *C. moschata*, and 666 *C. pepo* (https://seedvault.nordgen.org/) (Accessed 25 April 2021). The extent of overlap between different collections is generally not known. While potential duplications between collections can provide important backup, it also reduces total accessible diversity. It should also be noted that while collections can preserve diversity, they are also a snapshot in time, essentially a freeze-frame of evolutionary history at the time that the materials were collected [5]. 

The USDA NPGS, which distributes germplasm free of charge and restriction to scientists, educators, and producers, is a model for free exchange for crop species. Passport data in the Germplasm Resources Information Network (NPGS GRIN) system including information on collection date, location, collector, donor, donor date, and taxonomic classification can be obtained from the GRIN-Global website (https://npgsweb.ars-grin.gov/gringlobal/search.aspx) (Accessed 8 March 2021) [24]. Where available, GRIN-Global also includes phenotypic and evaluation data, images, and bibliographic citations of research papers with links to lists of accessions included in the publication. Some other collections, such as NARO, Japan, also have readily searchable databases and provide samples to researchers, but many germplasm collections and/or their data are difficult to access. Restricted interchange of germplasm among collections is a concern for publicly funded breeding programs, as countries have become more protective of their genetic resources [25]. Interestingly, international seed companies with active breeding programs within a given country may have access to germplasm that public breeders do not. Over time, this access may result in resource transfer among countries via trade and, eventually, into public breeding programs or programs of other companies if utility patents are not used for intellectual property protection or have expired. 

While major grain and legume crops may have tens- or even hundreds-of-thousands of accessions [23,26], collections of cucurbits are orders of magnitude smaller, with a few hundred or thousand per collection. This is, in part, due to the lower priority placed on the conservation of horticultural crops relative to grain and legume crops, the large number and variety of horticultural crops, and the frequently greater costs and technical difficulties associated with their collection and maintenance. Additional key factors influencing collection size and accessibility include size and diversity of historical collections, limited resources for necessary maintenance, the absence of essential characterization/evaluation data, phytosanitary issues associated with distributions, and limited funding for and access to opportunities to conduct or participate in collection expeditions. Not unique to the Cucurbitaceae, new acquisitions have been extremely limited in recent years due to difficulties securing permission for plant exploration and collecting in the regions of interest, driven in part by concerns for bioprospecting [25]. This is especially relevant in the presence of ongoing efforts to standardize germplasm management practices and consolidate germplasm conservation efforts internationally while ensuring that these resources remain globally available and are conserved in an efficient manner. In addition, barriers to acquisitions are particularly high for institutions that stipulate that germplasm within their collections will be freely distributed. As a result, most new acquisitions to open access institutions such as those administered by the USDA-NPGS have been from discontinued breeding programs, expired PVPs, or old cultivars no longer being conserved by seed companies, and materials collected within the continental US and its territories. 

Maintaining a collection requires appropriate storage conditions, regular viability testing, and regeneration of seed supplies when seed numbers or percent viability is low. Risks include loss of viability and genetic erosion through nonrandom viability during regeneration [27]. Biological characteristics of the Cucurbitaceae (i.e., large, insect-pollinated, outcrossing plants with spreading vines) that may have contributed to increased genetic bottlenecks associated with domestication, also make them expensive to regenerate and maintain. In addition, many of the wild accessions exhibit daylength sensitivity for flowering. These characteristics, combined with the necessity to perform seed regenerations in the field using enclosed cages with pollinators (e.g., honeybees, alfalfa leaf cutting bees, bumble bees), or by hand pollination in the greenhouse, further contribute to expenses and labor. As many PI accessions are genetically heterogeneous in nature, it is also a goal to prevent loss of the genetic diversity within each accession during regeneration efforts. Maintaining health and developing strategies to screen, prevent, and treat diseases during the regeneration process is also a vital concern. Healthy seed stocks free of seed-borne diseases are essential to preserve diversity and to meet disease-free import requirements for international distribution. As a result of these various constraining factors including cost, time, space, labor, and disease control, the total recorded number of accessions may not equal “active,” i.e., available, collections, further limiting accessible diversity. The importance of ex situ collections for ensuring future food security and responding to increasing environmental challenges is also recognized by Global Crop Diversity Trust (https://www.croptrust.org/project/conservation-strategies/) (Accessed 27 May 2021) [28], whose mission is to preserve crop diversity. Their work includes the development of global crop conservation strategies by examining available germplasm accessions (what is present and missing), determining resource needs for each crop species and how they are conserved, and determining priority actions needed to strengthen conservation. New strategies are currently under development for ten new crops/crop groups, including one for Cucurbitaceae species. 

## 2. Cucurbit Crops and Vulnerabilities

### 2.1. Melon (C. melo) 

*C. melo* includes a remarkably diverse group of herbaceous, annual plants of commercial importance worldwide. They are best known for their sweet, dessert fruits, primarily eaten fresh, but bitter or bland fruits are also consumed as vegetables, and in some countries (e.g., India and Turkmenistan), the seeds are eaten. Small-fruited agrestis types are dried in some areas, e.g., India, for use in soups when food is scarce [29]. The flesh of Waharman-type melon (Group Ameri) may be sliced and dried for consumption during the winter months in Turkmenistan [30]. The fruit flesh is a source of potassium, β carotene, and vitamin C (ascorbic acid) but is low in other nutrients, e.g., vitamin E, folic acid, iron, and calcium [31]. Content of ascorbic acid, folic acid, and potassium are influenced by the environment, e.g., soil type, and there appears to be potential for improvement in orange flesh cantaloupe and green fleshed honeydew [31,32,33]. In countries where seeds are eaten, they provide high-quality oil and proteinaceous meals and may be found in local markets as a component of “roaster mixes,” along with other cucurbit seeds [30,34].

#### 2.1.1. Melon Vulnerabilities

Melon producers face challenging and changing disease and insect pressures, along with climate change and water-related challenges. A summary in the form of a questionnaire to melon researchers listed 10 fungal diseases, 3 bacterial diseases, 33 viruses, and 8 insect pests [35]. Additional viruses, *Cucurbit chlorotic yellows virus* (CCYV) and *Squash vein yellowing virus* (SqVYV), have since been added. Cucurbit powdery mildew (CPM), incited by three fungal species, is the most ubiquitous disease in melons. Many races of one CPM pathogen, *Podosphaera xanthii* (Px), have been reported worldwide [36]. Research on host plant resistance to CPM incited by Px has been underway for nearly a century [37]. Sweet potato whitefly (*Bemisia tabaci*) also has emerged as a major pest worldwide over the past 30 years. In addition to vectoring several viral diseases, e.g., *Cucurbit yellow stunting disorder virus* (CYSDV), the sweet potato whitefly can devastate melon crops through feeding damage alone [38,39,40]. Water availability, droughts, and increased salinity are also potential long-term problems for melon production. To date, however, limited breeding has been conducted for resistance to abiotic stresses.

While the genetic base of melons is varied, commercial production favors distinct types in different parts of the world and market types appear to be stable. For example, US production is dominated by orange flesh cantaloupe and green flesh honeydew. The US western shipper-type cantaloupe fruits are known for their climacteric character, with harvest maturity indicated by the exterior color change of the fruit and the development of an abscission layer separating the fruit from the vine. Several harvests are often required to maximize yield. A large proportion of the modern US western shipper-type cantaloupe cultivars are based on the multiple disease-resistant ‘PMR 45’, which was developed by a cross of ‘Hale’s Best’ with California 525, a landrace from India, followed by one backcross to Hale’s Best and inbreeding for two generations [41,42]. The next generation of cultivars was based on ‘Top Mark’, which was developed by a commercial breeder. More modern versions, primarily F_1_ hybrids, produce larger fruit that set and mature earlier than Top Mark types. In recent years, there has been gradual adoption of Harper-type melon, e.g., ‘Caribbean Gold’, in the United States, Mexico, and Central America. Harper-type were developed for long shelf-life, which gives growers increased latitude in harvest timing to approach once-over harvest, albeit at the perceived loss of textural quality. 

The Harper-type long shelf-life cultivars have introduced new genetic variation into melon production areas; however, genetic erosion is a concern for the future of melon breeding. The overall trend worldwide is toward reduced variation. Melons produced in the eastern US, which were typically grown for local markers and marked by softer flesh and more intense musky flavor, are becoming replaced by newer hybrids more similar to the western shipper types. As farmers in developing countries seek more productive, uniform, and higher quality fruit, landraces or farmer varieties are gradually lost [29].

#### 2.1.2. Melon Genetic Resources

The US NPGS *C. melo* collection, maintained at the North Central Regional Plant Introduction Station in (NCRPIS) in Ames, Iowa, is the largest, publicly available melon germplasm resource. It includes 3228 accessions from more than 80 countries, of which 1936 accessions are available for distribution. In addition to *C. melo*, the collection includes 318 accessions from 22 additional *Cucumis* species from 31 countries, two of which are cultivated—*C*. *anguria* and *C. metuliferus*. The National Laboratory for Germplasm Resources Preservation (NLGRP) has a backup collection of 2605 *C. melo* accessions and 210 additional *Cucumis* accessions. Melons of African origin, a secondary center of diversity [14] are underrepresented in the NPGS melon collection. The Japanese collection lists 824 accessions, the majority of which can be requested for distribution. Other large collections of *C. melo* not recorded in the GeneSys database (Table 2), are maintained in Uzbekistan with 1330 accessions (the Uzbek Research Institute of Plant Industry, the Uzbek Research Institute of Vegetables, Melons, and Potato, the Karakalpak Research Institute of Agriculture, Tashkent [43]), China (CAAS—Chinese Academy of Agricultural Sciences), and India (ICAR—National Bureau of Plant Genetic Resources (NBPGR), New Delhi URL: nbpgr.ernet.in)), which is notable for its wealth of melon germplasm. However, many of these collections and their associated data are not readily accessible. Contamination with the seed-transmitted bacterial fruit blotch pathogen (*Acidovorax citrulli*) also endangers melon collections, and in the US, has reduced the numbers of accessions that are available for distribution. 

The global melon research community has extensively used the NPGS melon collection, especially for resistance to diseases, insects, and abiotic stresses. Evaluation data sets available on GRIN-Global for *C. melo* include anthracnose (*Colletotrichum lagenarium*), bacterial wilt (*Erwinia tracheiphila*), downy mildew (*Pseudoperonospora cubensis*), fusarium rot (*Fusarium solani* f. *cucurbitae*), leaf blight (*Alternaria cucumerina*), leaf spot (*Cercospora citrullina*), Macrosporium leaf blight (believed synonymous with *Alternaria cucumerina*), powdery mildew (*Podosphaera xanthii* [syn. *Sphaerotheca fuliginea*]), stem blight (*Mycosphaerella citrullina*), verticillium wilt (*Verticillium dahlia*), gummy stem blight, and root-knot nematode (*Meloidogyne incognita acrita*). Consistent with the extensive genetic diversity associated with the center of domestication, germplasm from India has proven to be a treasure trove of biotic and abiotic stress resistance, e.g., host plant resistance to downy and powdery mildews, Alternaria, Anthracnose, and aphid- and whitefly transmitted viruses [41,44,45]. 

### 2.2. Cucumber (Cucumis sativus) 

Cucumbers are an important vegetable consumed throughout the world. FAO statistics indicate that in 2019, more than 130 countries planted cucumber on 2.23 million hectares. Cucumber fruit, which are generally eaten when immature, are harvested at an early stage of fruit development. While less morphologically variable than *C. melo*, different kinds of cucumbers are produced depending on regional preferences and purposes. The two types most typically grown in the US and Europe serve distinct purposes. Processing or pickling cucumbers have comparatively short fruit (5 to 15 cm long) with thin, mottled, warty skin that is permeable to brining salt. Fresh-market, or slicing, cucumbers have somewhat longer fruit (20 to 23 cm) and smooth, uniformly green, thick skin. Long-fruited, parthenocarpic greenhouse cucumbers (30–40 cm), and mini (12–15 cm) cucumbers (Beit Alpha or Mediterranean cucumber, with very short, parthenocarpic fruit), are also grown for the fresh market. Cucumbers in east Asia, which are eaten fresh, typically have very long fruits (40–50 cm). Three major market classes, north China, south China, and Japanese, vary in appearance (warts, ribs), flavor, and crispness [19,46,47]. A semiwild botanical variety of cucumber, *C. sativus* L. var. *xishuangbannanesis*, is also cultivated in some subtropical parts of Asia (southwest China, Thailand, Laos, Myanmar) [47,48,49]. *Xishuangbannanesis,* which exhibits some unique traits including very large fruits (up to 5 kg), and orange flesh color due to accumulation of β-carotene, is eaten both immature and mature.

#### 2.2.1. Cucumber Vulnerabilities

In 2014, a survey of the global cucumber community was conducted to identify breeding needs [50]. Several critical issues common to major market classes of cucumbers have been identified, including resistances for downy mildew (*Pseudoperonospora cubensis*), *Cucurbit yellow stunting disorder virus* (CYSDV), and *Cucumber green mottle mosaic virus* (CGMMV). Priority issues for specific market classes or geographic regions of cucumber production have also been identified. For North American pickling and slicing cucumbers, top research priorities included resistances to the post-2004 downy mildew strains, Phytophthora fruit rot (*Phytophthora capsici*), and angular leaf spot (*Pseudomonas lachrymans*). Other economically important diseases worldwide include anthracnose (*Colletotrichum lagenarium*), Fusarium wilt (*Fusarium oxysporum* f. sp. *cucumerinum*), gummy stem blight (*Didymella bryoniae*), scab (*Cladosporium cucumerinum),* target leaf spot (*Corynespora cassiicola*), and viruses including *Cucumber mosaic virus* (CMV) and several potyviruses [51]. Changing production systems may also pose new challenges. For example, there is a growing trend in the greenhouse production of cucumbers for which CGMMV is becoming a major problem. Accessions from the US NPGS collection also have been recently distributed to screen for sources for heat and drought tolerance, indicating efforts to address vulnerabilities to abiotic stresses.

The demand by growers, buyers, and processors for uniformity in plant type, fruit type, and flowering time, combined with the need for resistance to the major disease problems in each production area, has restricted genetic diversity. In the US, a phenotypic and molecular survey of available cultivars has indicated that many are similar in parentage [52]. Many of the pickling cucumber hybrids have Gy14 as a significant source of germplasm in the seed parent and ‘Sumter’ and M21 as the pollen parent. The same is true for fresh-market cucumbers, where much use is made of ‘Marketmore’ and ‘Poinsett’, either directly or as a component of a hybrid. This concern is magnified if disease resistances are from a single source. The resulting cultivars will be more vulnerable to virulence changes of pathogens in the field as has occurred for downy mildew (DM) resistance. Almost all US cucumber varieties carry the *dm1* locus originating from PI 197087, which was very effective in protecting cucumbers from DM infection for nearly four decades [53,54]. However, in 2004, a new DM strain emerged which rendered *dm1* less effective. The breakdown has devastated the cucumber industry, emphasizing the importance of diversifying sources of disease resistance. 

#### 2.2.2. Cucumber Genetic Resources

In addition to genetic constraints imposed upon cucumber breeding by market demands, *C. sativus* is also genetically isolated from other *Cucumis* species, as it is the only *Cucumis* with 2n = 14 chromosomes [47]. The *C. sativus* species have been described to include four cross-compatible botanical varieties: the cultivated var. *sativus*; wild cucumber var. *hardwickii* (Royle) Alef; semiwild var. *xishuangbannanesis* Qi et Yuan [55], and var. *sikkimensis* Hook [56] (Sikkim cucumber). *Sativus*, *xishuangbannanesis,* and *sikkimensis* all share several chromosomal inversions and translocations in relationship to wild var. *hardwickii*, suggesting chromosomal changes during domestication [57,58]. The semiwild xishuangbannanesis cucumber is thought to have resulted from the subsequent diversifying selection of primitive cultivated cucumbers [59] and more recent genetic analyses suggest that the Sikkim cucumber should be considered as an ecotype of *C. sativus*, not worthy of formal taxonomic recognition [60].

Several countries maintain sizable collections of cucumber (e.g., countries listed in Table 2; China National Vegetable Germplasm Bank [26]; NARO Japan [61]), including 1334 *C. sativus* accessions available from the NCRPIS in Ames, Iowa, US. Diversity within cultivated *C. sativus* has provided numerous important traits of use in commercial breeding programs [62,63,64]. For example, CMV resistance derived from ‘Chinese Long’ virtually saved the processing industry in the midwestern US in the 1950s, and deployment of the anthracnose resistance gene from PI 197087 from India allowed a fall cucumber crop season in the southwest. The incorporation of the gynoecious sex expression gene from PI 260860 from Korea made it possible for large-scale production of commercial F_1_ hybrids and the development of once-over machine harvest systems. More recently, several PIs have been identified with resistance to the post-2004 strain of downy mildew [52,53,65,66]. Other traits identified in the collection include resistances to powdery mildew, Phytophthora fruit rot, and root-knot nematodes [67,68,69,70]

Numerous investigations have indicated that *xishuangbannanesis* and *hardwickii* cucumbers also possess valuable tolerances to biotic and abiotic stresses such as target leaf spot, gummy stem blight, and root-knot nematode (*Meloidogyne javanica*) [49,57,59,71,72,73]; however, publicly available germplasm is very limited for these wild and semiwild cucumbers. The only other species that has been successfully crossed with cucumber is *C. hystrix* (2n = 24), although with limited success as the chromosomal differences cause significant barriers to obtaining viable seed [74,75]. Development of a hybrid amphidiploid, *C. x hytivus* (2n = 4x = 38) as a bridge species allowed for increased cross-compatibility with *C. sativus*, raising the potential for eventual introgression of valuable traits [76]. These studies underscore the importance of the further acquisition of accessions of *C. sativus* vars. *hardwickii* and *xishuangbannanesis,* and *C. hystrix,* as they may harbor unique genes important for cucumber improvement.

### 2.3. Watermelon (C. lanatus) 

Watermelon, *C. lanatus*, is a monoecious, warm climate, annual plant with a vining growth habit that requires a long growing season. It is consumed throughout the world for its sweet, juicy fruits. The vast majority of the more than 100 Mt of watermelon fruit produced annually are sold fresh or freshly cut. However, it can also be eaten as jams, glazed candy, or pickled rinds [3,77]. Over the past 20 years, seedless watermelons have become increasingly popular, especially in the US, where they currently make up greater than 85% of production [78]. Consumers are also becoming more aware of watermelon as a source of nutritional compounds such as lycopene, citrulline, arginine, and vitamin C, which are associated with health benefits, including decreased risk of certain kinds of cancer, age-related degenerative pathologies, and heart disease [77,79]. Several watermelon juice products have entered the market, often promoted as sources of antioxidants and other health-promoting compounds. In some parts of the world, watermelons are also consumed for their seeds. *Citrullus mucosospermus*, a close relative of *C. lanatus*, is native to west Africa and has a modified fleshy mucilaginous seed coat that becomes paper-thin when dried. The thin seed coat makes it easier to de-hull the seed. In China, specific varieties of watermelon bred for edible seeds are produced on nearly a quarter-million hectares annually [3]. The edible-seed watermelons can grow on marginal land and are drought tolerant, with small thin leaves, thin vines, and a large number of branches. 

#### 2.3.1. Watermelon Vulnerabilities

There are numerous current, emerging, and evolving pathogens, insects, and pests that threaten watermelon production [80]. Among the primary diseases challenging production in the US are Fusarium wilt (*Fusarium oxysporum* f. sp. *niveum*), gummy stem blight (*Stagonosporopsis* spp), anthracnose (*Colletochrichum spp.*), powdery mildew (*Podosphaera xanthii*) Phytophthora fruit rot (*Phytophthora capsici*), bacterial fruit blotch (*Acidovorax citrulli*), and several viruses including the watermelon strain of *Papaya ringspot virus* (PRSV-W), *Zucchini yellow mosaic virus* (ZYMV), and *Cucumber green mottle mosaic virus* (CGMMV) [80,81]. Whiteflies and whitefly transmitted virus complexes have recently become an economically important constraint on watermelon production in the southeastern US [82,83]. There is also interest in sources of nematode-resistant germplasm for breeding and use as rootstocks [84,85]. Fluctuating environmental conditions associated with climate change that leads to drought stress will be especially challenging to crops such as watermelon that contains exceptionally high water content [6,86]. 

Preferred types of watermelon vary in different parts of the world. Most of the modern watermelon cultivars in China, which are characterized by round fruits with a thin rind, are derived from local, older cultivars crossed with cultivars introduced from Japan and the US [3,13]. In the US, watermelon production has transitioned from oblong, thick-rinded open-pollinated cultivars, to F_1_ hybrids, to seedless triploid hybrids that now dominate the market [78,87]. Seedless triploids are produced by crossing female tetraploid plants with diploid inbred lines as the male parent, resulting in sterile progeny. The production of triploid watermelons also has led to the development of diploid pollenizer cultivars that serve as the pollen source needed to stimulate fruit development [88]. Pollenizers are selected for compact growth habits, and small (often inedible) fruit that is not harvested, thereby reducing competition for space and resources with the fruit-bearing triploid plants. This complex breeding system and the resultant high costs of seed production have contributed to a shift in cultivar development from public breeding to private seed companies. Public institutions now most often focus on pre-breeding and release of improved germplasm for use in cultivar development by the private sector. Grafting watermelon scions to rootstocks of other cucurbit species also has become increasingly important as a method to provide resistance to soil-borne diseases. The very high levels of adoption of grafting in many parts of the world (>95% for intensely managed production in Japan, Korea, Greece, Israel, and Turkey) also provide an impetus for breeding rootstocks [89,90]. Despite these innovations, the necessity to retain desired combinations of horticultural and culinary characteristics, yield and agronomic performance, and adaptations to the production environment has resulted in a narrow genetic base for commercially produced watermelon cultivars [91,92]. Furthermore, genetic analysis indicates low genetic diversity for all *C. lanatus* accessions, despite different geographic origins of these accessions, likely due to strong bottlenecks during domestication [93].

#### 2.3.2. Watermelon Genetic Resources

Among the germplasm listings in GeneSys are large collections of *Citrullus* in Brazil, the US, Sudan, and Spain (Table 2). Additional significant collections are maintained in China and Russia. The NI Vavilov Research Institute of Plant Industry (VIR; http://www.vir.nw.ru, accessed on 12 May 2021) (Russian Federation) maintains >3500 *Citrullus* accessions, including many collected in southern Africa [94]. Other collections include China (CAAS) with 1197 accessions (FAO, 2010), Japan (NARO), which lists 251 *C. lanatus* accessions along with extensive phenotypic data, and the University of Cukurova in Turkey with seed for several hundred diverse cultivars and landraces, representing both European and Asian types, that were collected throughout the country [95]. The US NPGS (GRIN) system includes 1878 *Citrullus* accessions, of which approximately 75% are available for distribution. Along with 1613 accessions of *C. lanatus*, the collection includes at least one accession of each of the other six species of *Citrullus* (*C. mucosospermus, C. amarus, C. colocynthis, C. rehmii, C. ecirrhosus* and *C. naudinianus*), including 151 of *C. amarus* and 75 of *C. mucosospermus* (Table 3). Pictures of fruit and seed are available for most of the accessions in GRIN and new images were added in 2017–2018. Screening studies have found the NPGS collection to carry sources of many important traits including resistances to anthracnose, bacterial fruit blotch, downy mildew, gummy stem blight, leaf spot, powdery mildew, verticillium wilt, *Papaya ringspot virus, Squash vein yellowing virus, Watermelon mosaic virus, Zucchini yellow mosaic virus*, and root-knot nematodes [96]. As diversity in *C. colocynthis* and *C. amarus* is much higher than in *C. lanatus* [97], they potentially provide important genetic resources for watermelon breeders. Importantly, *C. amarus* and *C. mucosospermus,* which are valuable sources of resistance to many diseases, can be readily crossed with *C. lanatus*, albeit with variable fertility [3,15]. 

Considering recent insights into the origin of watermelon [15,16,98,99], additional accessions should be acquired, especially for centers of diversity in the northeast and southern Africa. While *C. lanatus* germplasm from southern Africa is not currently considered endangered, there is value in increasing collections, as it provides important sources of disease resistances such as powdery mildew, Fusarium wilt and anthracnose, abiotic stress resistances such as drought tolerance, and novel fruit shape traits [94,100,101,102]. The outlook for conservation of *Citrullus* species in situ is uncertain, making the acquisition of additional germplasm an important goal for germplasm collections. 

### 2.4. Squashes and Pumpkins (Cucurbita pepo, Cucurbita moschata, Cucurbita maxima)

The genus *Cucurbita* includes several species grown for fruit that may be known as squashes, pumpkins, or gourds [103,104,105]. These fruit exhibit vast morphological diversity, both within and between species, and the boundaries between species, fruit type, and usage are often blurred. FAO production statistics record them collectively, showing 22.9 Mt of squashes, pumpkins, and gourds harvested from 1.5 million hectares worldwide. The three most widely produced species are *C. pepo*, *C. moschata*, and *C. maxima*. Of these, *C. pepo* is the most economically important and is split into two subspecies: *C. pepo* subsp. *Pepo*, and *C. pepo* subsp. *ovifera* [106,107]. The extensive variation in fruit shape, size, surface texture, and color has led to different morphotypes or cultivar groups within each species, forming different market classes and serving different culinary or other purposes (Table 4). Depending on the morphotype, fruit are consumed at an immature stage as summer squash (e.g., cocozelle, marrow, zucchini, crookneck, straightneck, scallop), harvested usually just a few days post-anthesis, or at a mature stage as winter squash. Winter squashes have the benefit of long shelf life, permitting them to be stored for long periods of time and to serve as staple foods in many countries. Many also have intense orange flesh with high content of carotenoids, providing an important source of provitamin A [108,109]. Colorful and fancifully shaped *Cucurbita* fruits are also often used as ornamentals, and the large edible seeds are frequently eaten or extracted for oil. 

#### 2.4.1. Squash and Pumpkin Vulnerabilities

Cucurbita crops face numerous biotic and abiotic challenges. An increasing and evolving list of diseases, especially viruses (e.g., *Zucchini yellow mosaic virus, Papaya ringspot virus, Watermelon mosaic virus, Squash leaf curl virus; Tomato leaf curl New Delhi virus, Cucumber mosaic virus, Cucurbit leaf crumple virus, Cucurbit yellow stunting disorder virus*), transmitted by aphids, whiteflies, and other insects are particularly devastating [110]. Whiteflies themselves also can cause major damage [111]. The need for resistance will continue to increase as climate change expands the natural range of herbivorous and disease-vectoring insects. Cucurbita species also suffer losses to bacterial, fungal, and oomycete diseases that plague other cucurbit crops such as powdery mildew (*Podosphaera xanthii* and *Golovinomyces cichoracearum*), downy mildew (*Pseudoperonospora cubensis*), Phytophthora capsici, gummy stem blight (*Didymella bryoniae*), and *Fusarium* spp. [105]. Even in cases where resistance has been incorporated, production security may be precarious. For example, powdery mildew is a ubiquitous pathogen of *Cucurbita*, but only a single dominant gene has widespread use to control the powdery mildew pathogen [112]. Abiotic challenges include adaptations to local environments and reducing days to maturity to fit volatile growing seasons. Future improvement of *Cucurbita* crops is constrained by limited investment in breeding. For example, despite multiple species and cultivar types, there are fewer than three full-time public *Cucurbita* breeding programs in the US. 

#### 2.4.2. Squash and Pumpkin Genetic Resources

While the *Cucurbita* species are native to different regions of the Americas [113], distribution and cultivation throughout the world have resulted in secondary centers of diversity in Europe and Asia: Europe for *C. pepo* var. *pepo* [114], India–Myanmar for *C. moschata* [115], and Japan–China for *C. maxima* [116]. In addition to intraspecific diversity, close genetic relationships among the three species may also provide opportunities for crop improvement. Breeding can involve interspecific crosses for trait introgression or progeny may be used directly as interspecific hybrids. *C. moschata* plays an important role as it is cross-fertile to various degrees with *C. pepo* and *C. maxima* and can thus be used as a bridge to move genes across species [115].

The largest *ex situ Cucurbita* collection, according to GeneSys records, is held by Brazil (National Genetic Resources Network (RENARGEN)), with more than 6000 accessions (Table 2). However, accessibility to these materials can be restricted by Brazilian laws limiting the export of, and research with, native germplasm [117]. Other large collections are maintained in the US, Ukraine, Spain, and the Tropical Agronomic Research and Teaching Center (CATIE). CATIE has a collection of 2119 *Cucurbita* collected from the Americas, including 1613 *C. moschata*, 174 *C. ficifolia*, 169 *C. pepo*, and 112 *C. argyrosperma* [118]. The US NPGS *Cucurbita* collections include representatives of 18 species, with the largest contributions from *C. pepo (980)*, *C. moschata (844)*, *C. maxima (838),* and *C. argyrosperma (278)*. Seed-borne diseases such as *Squash mosaic virus*, and daylength sensitivities, especially for many accessions of *C. moschata* which limit the ability to carry out seed regenerations, make only about half of the accessions available for distribution. 

The *Cucurbita* collections have been of great value to squash and pumpkin breeding for decades. Resistances to the potyviruses *Zucchini yellow mosaic virus* and *Papaya ringspot virus* have been utilized from *C. moschata* lines ‘Nigerian Local’ and ‘Menina’ [119]. Resistance to powdery mildew was originally introgressed from the wild species *C. okeechobeensis* subsp. *martinezii* into *C. moschata,* and later also into cultivars of *C. pepo* [112]. Recently identified resistance to *Phytophthora capsici* has been deployed in *C. pepo* breeding lines and shared with the seed industry [120]. Despite the clear value, the collection is limited in diversity; considering the variety of crops and number of species, there are very few accessions in the collection. Investment in collecting germplasm from diverse global cultures will be important to preserve valuable genetic diversity. 

## 3. Managing Cucurbit Genomic Resources

Germplasm management to ensure viability for the future is a common challenge across all crops. Several reports have emphasized the importance of documenting genetic diversity present within collections [4,5,27,121,122]. This effort is greatly aided by continually improving genomic, bioinformatic, and phenomic tools that enable the assessment of genetic structure and diversity within collections and facilitate comparisons among collections. These analyses can also identify gaps in collections or geographical regions that may be particularly important for further acquisitions or in situ protection. These analyses also provide the potential to establish genomically informed core populations or other tailored subsets to facilitate the identification, mapping, and utilization of valuable traits. While initially applied to crops such as wheat, maize, and soybean, continuous advances, and reductions in cost have made such technologies increasingly accessible to many minor crops, including cucurbits.

### 3.1. Documenting Genetic Diversity in Cucurbits

Several recent studies have performed genomic analyses of cucurbit accessions. Lv et al. [19] used a set of 23 simple sequence repeat markers (SSRs) to fingerprint 3342 cucumber accessions from Chinese, Dutch, and US collections, thereby providing information about genetic diversity and population structure within *Cucumis sativus*. Zhao et al. (2019) [12] mapped genetic variation in melon by resequencing a set of 1175 *C. melo* accessions assembled from Chinese and US collections (National Mid-term Genebank for Watermelon and Melon, Zhengzhou, China; Zhengzhou Fruit Research Institute, Chinese Academy of Agricultural Sciences; US NPGS). Whole-genome resequencing of 414 accessions representing the seven extant *Citrullus* species allowed for the reconstruction of the evolutionary history in this genus [97].

A systematic effort to genetically characterize the USDA NPGS collections for watermelon, melon, cucumber, and *Cucurbita* spp. was undertaken by the US CucCAP project [81]. All available *Cucumis sativus*, *C. melo*, *Citrullus lanatus*, and *Cucurbita pepo*, *maxima*, and *moschata* accessions were genotyped by sequencing (GBS) for each crop: 1234 for cucumber [20]; 1365 for watermelon [93]; 2083 for melon [123]; 829 for *C. pepo*; 534 for *C. maxima*; 314 for *C. moschata* (Hernandez et al., unpublished). The resulting data provided information about the nature and extent of relationships, genetic diversity, and population structure among the accessions within each collection as well as their relationship to geographical origin and morphotype. The genetic information was used to construct core populations of 380–400 PIs/crop representing 95–98% of the variation in each collection (for *Cucurbita*, the core population was designed for *C. pepo*). Efforts are underway to produce inbred lines, increase seed phenotype, and resequence the accessions within the core collections. These populations will provide genome-wide association study (GWAS) panels for further marker-trait association analyses. It is intended to make seeds of these inbred lines accessible for the scientific and breeding community.

### 3.2. Future Outlook

The prior crop sections emphasize the important role that genetic resources have played in cucurbit breeding and crop improvement. These genetic resources also harbor valuable natural variation for our fundamental understanding of unique biological phenomena in cucurbits such as sex expression, vascular structure, and fruit quality. Future efforts to preserve, increase and improve these resources should include renewed efforts to collect, both in centers of origin and regions with secondary sources of diversity such as southern Africa for watermelon, north and west Africa for melon, east Asia for cucumber, and south and east Asia for *Cucurbita moschata* and *maxima*, respectively. Opportunities for improvement of current collections also include the increased collection of related, interfertile species or wild relatives that can provide valuable novel sources of important traits such as *Citrullus amarus* and *C. mucosospermus* for watermelon, *Cucumis sativus* var. *hardwickii, xishuangbannanesis*, and *C. hystrix* for cucumber, and several *Cucurbita* species. Even wild species and relatives that are not interfertile may afford new opportunities to provide abiotic and biotic stress resistances by serving as rootstocks for grafted cropping systems. 

Further genetic analyses of collections around the world will enable the assessment of diversity within collections as well the opportunity to identify overlaps and gaps among collections. Along with these analyses, it is imperative that the resultant data be made available according to findable, accessible, interoperable, reusable (FAIR) principles [25,124]. USDA NPGS, in collaboration with Crop Trust and Biodiversity International, developed the GRIN-Global system to provide a plant genetic resource information management system [27]. While this and related efforts such as GeneSys allow for sharing of information of gene bank collections, they do not currently accommodate genomic data. Cucurbit genomic resources, including assembled genomes and annotations, genetic maps, transcriptomes, expressed sequence tag (EST), and genotyping by sequencing (GBS) data along with analysis and visualization tools are available through the CucCAP-associated Cucurbit Genomics Database (CuGenDB; http://cucurbitgenomics.org) (Accessed 7 March 2021) [125]. Future efforts will include the incorporation of linked phenotype data. The long-term viability of the database, however, poses a challenge. It will be important to efficiently integrate current and future genomic and phenotypic data to GRIN-Global to ensure that the data can be exploited to their full potential. Ultimately, the development of robust databases that allow for sharing of passport, genetic, genomic, and phenotypic data will facilitate more effective utilization of resources for crop improvement as well as physiological, ecological, and evolutionary studies. 

## 4. Conclusions

The Cucurbitaceae family is a rich source of nutritionally valuable crops with extensive diversity distributed throughout the world. Successful production of cucurbit crops is increasingly challenged by biotic and abiotic threats as pests and pathogens are moved from one part of the world to another and climate change alters local temperature and rainfall patterns. One of the most valuable resources to help manage these problems is genetic diversity. Preservation of this diversity, especially in the face of the ongoing loss of habitat of wild or feral populations, is imperative. Various national and international efforts to preserve diversity via ex situ collections provide tremendous resources that have been extensively utilized by the breeding community. However, many of these collections are challenged by limited resources, restricted access, and declining opportunities to expand collections to capture additional sources of diversity. While these challenges are not unique to cucurbits, the cucurbit community must continue to work with broader national and international efforts to assure that critical diversity is maintained, characterized, and made available for scientific inquiry and crop improvement.

## Figures and Tables

**Table 1:** Worldwide cucurbit crop production (Mtonnes) in 2019. Data are from the Food and Agriculture Organization of the United Nations, (http://www.fao.org/faostat/en/#data/QC) (Accessed 10 March 2021) [2].

Watermelon	Mt	%	Cucumber	Mt	%	Melons	Mt	%	Squashes, Pumpkins	Mt	%
Total worldwide	100.4		Total worldwide	87.8		Total worldwide	27.5		Total worldwide	22.9	
(128 countries)			(132 countries)			(105 countries)			(120 countries)		
top 12 producers	82.4	82	top 12 producers	79.4	90	top 12 producers	22.9	83	top 12 producers	16.1	70
China	60.7	60	China	70.3	80	China	13.5	49	China	8.4	37
Turkey	3.9	4	Turkey	1.9	2	Turkey	1.8	7	Ukraine	1.3	6
India	2.5	2	Russian Fed.	1.6	2	India	1.3	5	Russian Fed.	1.2	5
Brazil	2.3	2	Ukraine	1.0	1	Kazakhstan	1.0	4	Spain	0.7	3
Algeria	2.2	2	Iran	0.9	1	Iran	0.9	3	Mexico	0.7	3
Iran	1.9	2	Uzbekistan	0.9	1	Egypt	0.7	3	Bangladesh	0.6	3
Russian Fed.	1.8	2	Mexico	0.8	1	United States	0.7	3	United States	0.6	3
United States	1.7	2	Spain	0.7	1	Spain	0.7	3	Turkey	0.6	3
Egypt	1.6	2	United States	0.7	1	Guatemala	0.6	2	Italy	0.6	3
Mexico	1.4	1	Japan	0.5	1	Mexico	0.6	2	Indonesia	0.5	2
Kazakhstan	1.4	1	Poland	0.5	1	Italy	0.6	2	Cuba	0.4	2
Uzbekistan	1.2	1	Kazakhstan	0.5	1	Brazil	0.6	2	Algeria	0.4	2

**Table 2:** The major cucurbit germplasm collections recorded in the GeneSys system (https://www.genesys-pgr.org) [22] ^1^ (Accessed 25 April 2021).

**Cucumber (*Cucumis sativus*)**		**Melon (*Cucumis melo*)**	
Country	No. of accessions	Country	No. of accessions
US	1403	US	3954
Bulgaria	1030	Spain	1789
Netherlands	924	Brazil	654
Czechoslovakia	751	Germany	448
Germany	611	Ukraine	406
Poland	609	Hungary	252
Spain	521	Portugal	221
Taiwan	394	Taiwan	178
Ukraine	391	Poland	136
Hungary	265	Azerbaijan	114
**Watermelon** **(*C. lanatus//Citrullus* sp.)**		**Squashes/Pumpkins** **(*Cucurbita* spp.-*primarily pepo, moschata, maxima*)**	
Country	No. of accessions	Country	No. of accessions
Brazil	2007//2010	Brazil	6155
US	1922//2211	US	4635
Sudan	469//471	Ukraine	2114
Spain	428//435	Spain	1468
Ukraine	395//452	Taiwan	1115
Germany	249//266	Hungary	1088
Hungary	240//253	Germany	1058
Bulgaria	--//242	Portugal	887
Poland	101//101	Bulgaria	599

^1^ Other countries and germplasm repositories with significant cucurbit holdings not currently documented in GeneSys include those in China (China National Vegetable Germplasm Bank, Institute of Vegetables and Flowers at the Chinese Academy of Agricultural Sciences (IVF-CAAS)), Japan (National Agriculture and Food Research Organization (NARO); https://www.naro.affrc.go.jp/archive/nias/eng/genresources/index.html) (Accessed 20 April 2021), Russia (the Vavilov Institute of Plant Industry; http://www.vir.nw.ru) (Accessed 20 April 2021), India (Indian Council of Agricultural Research (ICAR)–National Bureau of Plant Genetic Resources (NBPGR) (http://www.nbpgr.ernet.in/) (Accessed 20 April 2021), Korea (National Agrobiodiversity Center, (http://genebank.rda.go.kr/) (Accessed 15 June 2021), Uzbekistan (the Uzbek Research Institute of Plant Industry, the Uzbek Research Institute of Vegetables, Melons, and Potato, the Karakalpak Research Institute of Agriculture, Tashkent, and the World Vegetable Center (https://avrdc.org, http://seed.worldveg.org/) (Accessed 15 June 2021)).

**Table 3:** Active *Citrullus* accessions in the NPGC. (https://npgsweb.ars-grin.gov/gringlobal/search.aspx) (Accessed 8 March 2021) [24].

Species	Number of Accessions
*C. amarus*	151
*C. colocynthis*	24
*C. ecirrhosus*	3
*C. lanatus*	1613
*C. mucosospermus*	75
*C. naudinianus*	7
*C. rehmii*	4
*Citrullus* spp.	1

**Table 4:** Commonly produced fruit morphotypes of *Cucurbita* species (sources: 103–105).

*C. pepo var. pepo*	Cocozelle ^1^, Pumpkin, Spaghetti squash, Vegetable Marrow ^1^ Zucchini ^1^
*C. pepo var. ovifera*	Acorn, Crookneck ^1^, Delicata, Scallop ^1^, Straightneck ^1^
*C. moschata*	Butternut, Cheese pumpkin, Japonica, Tropical (Calabaza)
*C. maxima*	Banana, Buttercup/Kobocha, Giant pumpkin, Hubbard, Kuri, Turban

^1^ Primarily eaten as summer squash.

## Data Availability

Not applicable.
